# Mass cytometry identifies characteristic immune cell subsets in bronchoalveolar lavage fluid from interstitial lung diseases

**DOI:** 10.3389/fimmu.2023.1145814

**Published:** 2023-03-06

**Authors:** Kentaro Hata, Toyoshi Yanagihara, Keisuke Matsubara, Kazufumi Kunimura, Kunihiro Suzuki, Kazuya Tsubouchi, Daisuke Eto, Hiroyuki Ando, Maki Uehara, Satoshi Ikegame, Yoshihiro Baba, Yoshinori Fukui, Isamu Okamoto

**Affiliations:** ^1^ Department of Respiratory Medicine, Graduate School of Medical Sciences, Kyushu University, Fukuoka, Japan; ^2^ Division of Immunogenetics, Department of Immunobiology and Neuroscience, Medical Institute of Bioregulation, Kyushu University, Fukuoka, Japan; ^3^ Division of Immunology and Genome Biology, Department of Molecular Genetics, Medical Institute of Bioregulation, Kyushu University, Fukuoka, Japan

**Keywords:** mass cytometry (CyTOF), idiopathic pulmonary fibrosis, connective-tissue disease-related interstitial lung disease, sarcoidosis, monocyte, FCRL5

## Abstract

Immune cells have been implicated in interstitial lung diseases (ILDs), although their phenotypes and effector mechanisms remain poorly understood. To better understand these cells, we conducted an exploratory mass cytometry analysis of immune cell subsets in bronchoalveolar lavage fluid (BALF) from patients with idiopathic pulmonary fibrosis (IPF), connective-tissue disease (CTD)-related ILD, and sarcoidosis, using two panels including 64 markers. Among myeloid cells, we observed the expansion of CD14^+^ CD36^hi^ CD84^hi^CCR2^–^ monocyte populations in IPF. These CD14^+^ CD36^hi^ CD84^hi^ CCR2^–^ subsets were also increased in ILDs with a progressive phenotype, particularly in a case of acute exacerbation (AEx) of IPF. Analysis of B cells revealed the presence of cells at various stages of differentiation in BALF, with a higher percentage of IgG memory B cells in CTD-ILDs and a trend toward more FCRL5^+^ B cells. These FCRL5^+^ B cells were also present in the patient with AEx-IPF and sarcoidosis with advanced lung lesions. Among T cells, we found increased levels of IL-2R^+^ TIGIT^+^ LAG3^+^ CD4^+^ T cells in IPF, increased levels of CXCR3^+^ CD226^+^ CD4^+^ T cells in sarcoidosis, and increased levels of PD1^+^ TIGIT^+^ CD57^+^ CD8^+^ T cells in CTD-ILDs. Together, these findings underscore the diverse immunopathogenesis of ILDs.

## Introduction

Interstitial lung disease (ILD) is a broad term for a group of disorders characterized by varying degrees of inflammation and scarring or fibrosis of the lung (1). In the majority of cases, ILD is a chronic disease with progressive scarring of lung tissue. Idiopathic pulmonary fibrosis (IPF) is the most common form of ILD and has a median survival rate of 3–5 years (2). Connective tissue disease (CTD), defined as systemic disorders characterized by autoimmune-mediated organ damage and circulating autoantibodies, is one of the common systemic diseases associated with ILD (3). Sarcoidosis is a granulomatous disorder of uncertain etiology, impacting various organ systems. Pulmonary involvement, including ILDs, represents the majority of morbidity and mortality associated with sarcoidosis (4).

It has been suggested that immune cells are involved in the pathogenesis of ILDs (4–7), with various subsets of immune cells potentially contributing to the development of ILDs, particularly macrophages and lymphoid cells. Macrophages, the most abundant immune cells in the lungs (8), have been shown to play a key role in the initiation and progression of fibrotic responses (9). The ability of macrophages to alter their functional traits in response to external stimuli allows them to exhibit a range of biological impacts. Recent studies have shown the existence of a unique population of macrophages in IPF lung explants (10–12) and murine models of pulmonary fibrosis (13, 14). T cells have also been implicated in the development of ILDs (6, 15). In addition, increasing evidence suggests that pathogenic B cells may contribute to the development of autoimmune diseases (16–18), although their role in the development of ILDs remains poorly understood. The inability to fully characterize immune cells in clinical specimens is partially due to a deficiency in available assays. While next-generation sequencing analysis has facilitated the thorough analysis of cells, there is a constraint on the number that can be examined. It can be challenging to accurately classify the type of immune cells solely based on transcripts. Mass cytometry, on the other hand, offers an in-depth, high-dimensional description of the immune cell population through the simultaneous utilization of multiple markers (19), which will significantly alter our understanding of ILDs.

The objective of this study is to utilize mass cytometry to phenotype macrophages, B cells, and T cells in bronchoalveolar lavage fluid (BALF) cells from patients with IPF, CTD-ILD, and sarcoidosis. By employing both unbiased and manually classified methods, we functionally characterize subpopulations of myeloid cells, B cells, and T cells that are overexpressed in each disease, thereby identifying cells that may potentially affect disease progression.

## Materials and methods

### Patients

**Figure 1:**
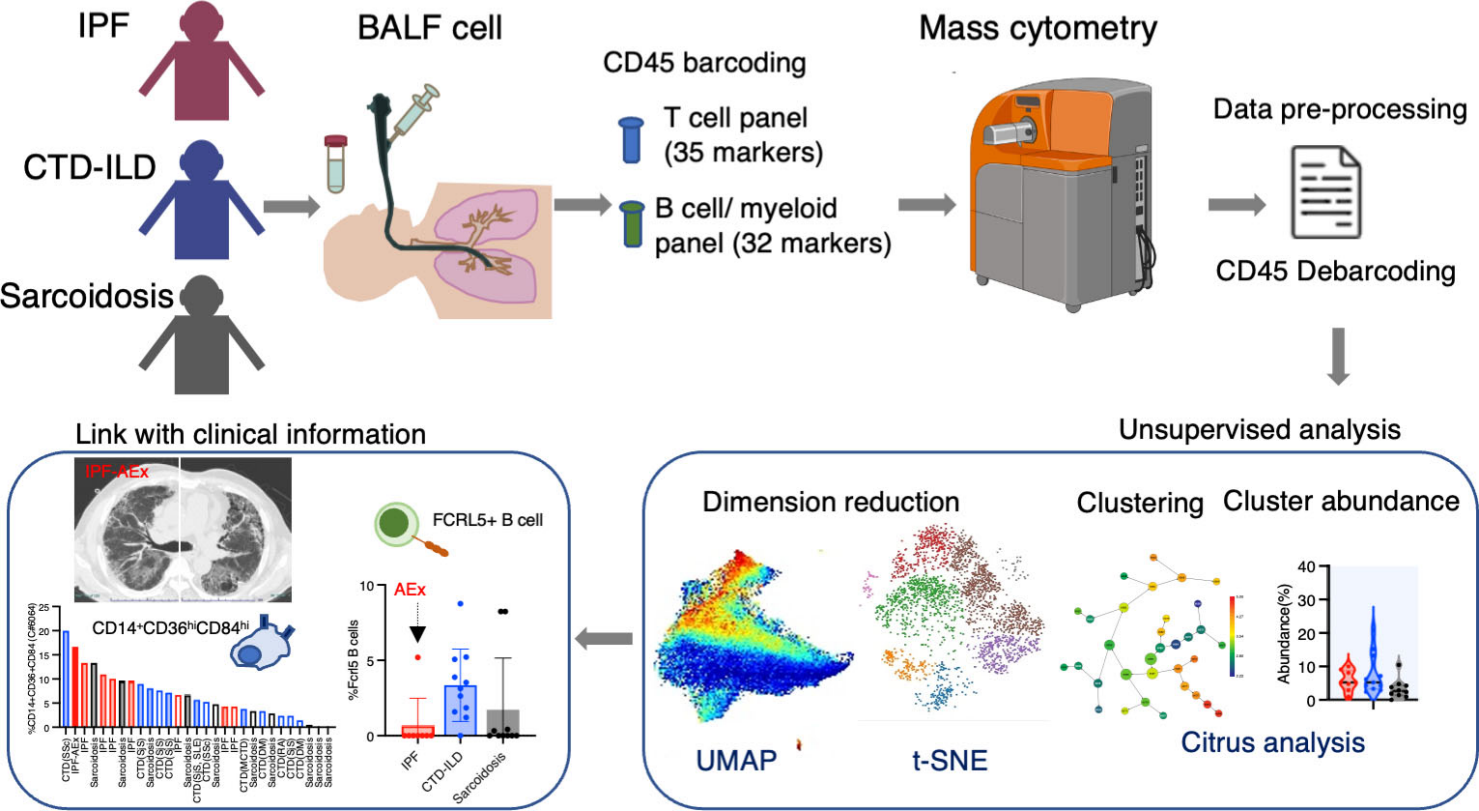
Graphical abstract of the study. Bronchoalveolar lavage fluid (BALF) samples were collected from patients with idiopathic pulmonary fibrosis (IPF), connective-tissue disease (CTD)-related ILD, and sarcoidosis. Following CD45 barcoding for individual sample identification, BALF cells were analyzed with a T cell panel (35 markers) and B cell/myeloid cell panel (32 markers) using mass cytometry. The expansion of CD14^+^ CD36^hi^ CD84^hi^ monocyte populations was found in IPF and ILDs with a progressive phenotype. FCRL5^+^ B cells were increased in CTD-ILDs, acute exacerbation (AEx) of IPF, and sarcoidosis with advanced lung lesions.

Patients who underwent BALF collection and were newly diagnosed with IPF, CTD-ILD, and sarcoidosis between Jan 2017 and April 2022 at Kyushu University Hospital were eligible for enrollment in the study. The study was authorized by the Ethics Committee of Kyushu University Hospital (reference number 22117-00). The diagnostic criteria for IPF, CTD-ILD, and sarcoidosis were as described elsewhere (20–24). Interstitial pneumonia with autoimmune features (IPAF) was included in CTD-ILD in this study. Disease progression was defined as follows: a relative decline of at least 10% in the predicted value of forced vital capacity (FVC), a relative decline of 5-10% in the predicted value of FVC accompanied by worsening respiratory symptoms or increased lung involvement on high-resolution CT imaging, or worsening respiratory symptoms and increased lung involvement within 24 months, as modified by the inclusion criteria of the INBUILD trial (25). The experimental and analytical workflow is shown in **Figure 1**.

### Mass cytometry

Antibodies were purchased either already metal-tagged (Standard Biotools) or in purified form (**Supplementary Table 1**). Purified antibodies were conjugated with metals using the Maxpar Antibody Labeling Kit (Standard Biotools) according to the manufacturer’s instructions and stored at 4°C. The cell labeling was performed as previously described (26). Briefly, cryopreserved BALF cells in Cellbanker1 (Takara #210409) were thawed in PBS and stained with Cell-ID™ Cisplatin-198Pt (Standard Biotools #201198, 1:2000 dilution) in PBS. Cells were incubated with FcR blocking reagent (Miltenyi, #130-059-901) and barcoded with each metal-labeled CD45 antibodies (**Supplementary Table 1**). After washing, CD45-labelled cells were mixed (maximum 6 samples together) and stained with APC-conjugated FCRL5 antibodies (BioLegend #340305)(for panel #2), followed by staining antibody cocktail (Panels #1 and #2, see **Supplementary Table 1**). The volume of antibodies was determined by preliminary experiments with metal minus one. After staining, cells were washed, fixed with 1.6% formaldehyde, and resuspended in Cell-ID Intercalator 103Rh (Standard Biotools #201103A) in Fix and Perm buffer (Standard Biotools) at 4°C overnight. For acquisition, cells were resuspended in MaxPar Cell Acquisition Solution (Standard Biotools #201240) containing one-fifth EQ Four Element Calibration Beads (Standard Biotools #201078) and were acquired at a rate of 200–300 events/second on a Helios mass cytometer (Standard Biotools). Files were converted to FCS, randomized, and normalized for EQ bead intensity using the Helios software. Concatenating fcs files in the same group into one file was conducted by FlowJo v10.8 (BD Biosciences). Manual gating, visualization of t-distributed stochastic neighbor embedding (tSNE), Uniform manifold approximation and projection (UMAP) analysis, and Citrus analysis (27) were performed using Cytobank Premium (Cytobank Inc.).

### Data analysis

Live cells were selected by the exclusion of cisplatin-positive cells and doublets. CD45^+^ were selected and further analyzed. We first conducted principal component analysis (PCA) using median expression as a univariate summary for each marker on total cells, myeloid cells (CD11b^+^CD11c^+^), and T cells (CD2^+^CD3^+^) in the three diseases of interest (IPF, CTD-ILD, and sarcoidosis) using R package *factoextra, ggplot2, ggplotly* in RStudio (version 2022.12.0 + 353 with R version 4.2.2) to compare samples at a gross level (**Supplementary Figure 1**). For myeloid cells, CD11b^+^CD11c^+^ cells were gated, and the Citrus algorithm was conducted with clustering channels of CD11b, CD11c, CD64, CD14, CD16, CD32, CD36, CD38, CD84, CD86, CD163, CD206, CD209, CD223, HLA-DR, CCR2, CCR5, ST2 using following parameters: association models = nearest shrunken centroid (PAMR), cluster characterization = abundance, minimum cluster size = 5%, cross-validation folds = 5, false discovery rate = 1%. Two cases of CTD-ILDs (dermatomyositis-related, IgG4-related) were excluded due to low cell numbers to calculate clusters. A UMAP analysis was performed on myeloid cells, incorporating clustering channels of CD11b, CD11c, CD64, CD14, CD16, CD206, HLA-DR, and CCR2 with the following parameters: numbers of neighbors = 10, minimum distance = 0.01 (comparison between IPF, CTD-ILD, and sarcoidosis), or with clustering channels of CD11b, CD11c, CD64, CD14, CD16, CD32, CD36, CD38, CD84, CD86, CD163, CD206, CD209, CD223, TIM-1, HLA-DR, CCR2, CCR5, ST2 using following parameters: numbers of neighbors = 15, minimum distance = 0.01 (comparison among IPF cases).

For B cells, CD3^–^CD64^–^ and CD19^+^ or CD138^+^ cells were gated, and viSNE was conducted with clustering channels of CD19, CD38, CD11c, IgA, IgG, CD138, CD21, ST2, CXCR5, CD24, CD27, TIM-1, IgM, HLA-DR, IgD, FCRL5 on individual files and concatenated files using following parameters: iterations = 1000, perplexity = 30, theta = 0.5. Two cases of CTD-ILDs (dermatomyositis-related, IgG4-related) were excluded due to low cell numbers to calculate clusters.

For T cells, the CD2^+^CD3^+^ cell population was selectively gated in concatenated files. A UMAP analysis was performed on T cells, incorporating clustering channels of CD4, CD8, CD45RA, CD45RO, CCR7, CD28, and Fas, with the following parameters: numbers of neighbors = 10, minimum distance = 0.01. The Citrus algorithm was conducted with clustering channels of CD4, CD5, CD7, CD8a, CD11a, CD16, CD27, CD28, CD44, CD45RA, CD45RO, CD49d, CD57, CD69, CD226, Fas, IL-2R, PD-L1, PD-L2, PD-1, OX40, TIGIT, TIM3, CTLA-4, CD223 (LAG-3), BTLA, ICOS, ST2, CCR7, CXCR3, HLA-DR using following parameters: association models = nearest shrunken centroid (PAMR), cluster characterization = abundance, minimum cluster size = 5%, cross-validation folds = 5, false discovery rate = 1%. viSNE for T cells was conducted with clustering channels of CD4, CD5, CD7, CD8a, CD11a, CD16, CD27, CD28, CD44, CD45RA, CD45RO, CD49d, CD57, CD69, CD226, Fas, IL-2R, PD-L1, PD-L2, PD-1, OX40, TIGIT, TIM3, CTLA-4, CD223 (LAG-3), BTLA, ICOS, ST2, CCR7, CXCR3, HLA-DR using following parameters: iterations = 1000, perplexity = 30, theta = 0.5. Choosing between UMAP and viSNE as the dimensionality reduction tool was based on their ability to preserve the connections between global structures and the distances between cell groups (UMAP was better than viSNE) and their ability to display a clear, non-overlapping representation of cell subgroups that makes it easier to see the differences between groups (viSNE was better than UMAP).

Heatmaps were generated using the R package *ComplexHeatmap* in RStudio by calculating the median expression levels of each channel within the Citrus-generated clusters, normalizing the values to a maximum of 100 to denote maximum expression. The numerical values of total cells, T cells, myeloid cells, and B cells in each case are shown in **Supplementary Table 2**.

### Statistical analysis

For the Citrus algorism experiment, we employed a PAMR association model, using a stringent threshold of 1% FDR, as outlined in the *Data Analysis* section. The Student’s two-tailed unpaired t-test was utilized to perform a comparative analysis between the two groups. The statistical analysis was conducted through a one-way ANOVA, complemented by Tukey’s multiple comparison tests, to determine the significance among the three groups. A two-way ANOVA, accompanied by Tukey’s multiple comparison tests, was performed to evaluate the manually gated cell proportions using GraphPad Prism 9 software. A P-value less than 0.05 was considered to indicate statistical significance.

## Results

### Patient characteristics

We analyzed 8 cases of IPF, 13 of CTD-ILD, and 10 of sarcoidosis (**Table 1**). Among the IPF cases, one patient experienced acute exacerbation (AEx) of IPF on admission. Thirteen cases of CTD-ILDs were observed, comprising Sjogren’s syndrome (SjS) (n = 3), dermatomyositis (DM) (n = 3), systemic sclerosis (SSc) (n = 2), mixed connective tissue disease (MCTD) (n = 1), systemic lupus erythematosus (SLE) complicated with adult-onset Still’s disease (AOSD) (n = 1), IgG4-related disease (n = 1), rheumatoid arthritis (RA)(n = 1), and IPAF (n = 1). The presence of autoantibodies and radiological patterns in each of the CTD-ILD cases and the diagnosis of the IPAF case were described in **Supplementary Table 3**.

**Table 1:** Characteristics of the study population.

	IPF	CTD-ILD	Sarcoidosis
Number	8	13	10
Age	72.0 ± 7.6	66.2 ± 15.1	48.7 ± 15.2
Male	100 (8)	38.5 (5)	60 (6)
Pack-years	26.2 ± 28.8	15.5 ± 19.5	13.7 ± 24.3
BALF differential cell counts (%)			
Macrophage	82.2 ± 10.5	65.1 ± 25.7	55.6 ± 29.9
Neutrophil	6.2 ± 3.9	7.5 ± 18.1	1.9 ± 2.7
Lymphocyte	9.5 ± 9.4	26.0 ± 23.2	42.0 ± 31.0
Eosinophil	2.0 ± 1.5	1.4 ± 1.5	0.6 ± 0.5

Data for age and BALF analysis are presented as mean ± SD. IPF, idiopathic pulmonary fibrosis; CTD-ILD, connective tissue disease-related ILD; BALF, bronchoalveolar lavage fluid.

Differential cell counts for BALF revealed lymphocytosis in 1 out of 8 cases (12.5%) of IPF as well as in 7 out of 13 cases (53.8%) of CTD-ILD and 7 out of 11 cases (63.6%) of sarcoidosis when the cut-off for the percentage of lymphocytes was set to >20%.

### Expansion of CD14^+^CD36^hi^CD84^hi^ *CCR2^–^* monocytes in BALF from patients with IPF

**Figure 2:**
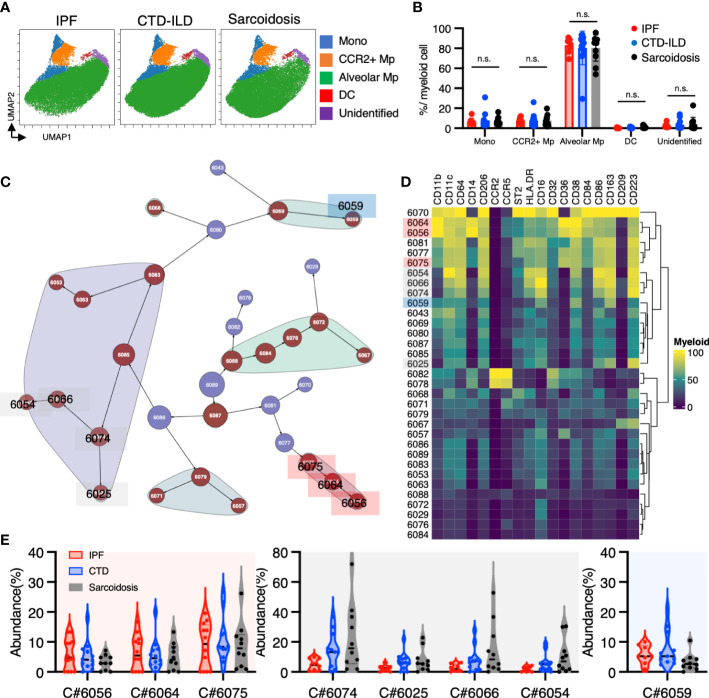
Characterization of myeloid cell subsets in BALF from patients with IPF, CTD-ILD, and sarcoidosis. **(A)** UMAP plots of concatenated samples visualizing the distribution of CD11b^+^CD11c^+^ myeloid cell subpopulations in BALF from patients with IPF, CTD-ILD, and sarcoidosis. Monocytes are defined by CD64^+^CD14^+^, CCR2^+^ macrophages (Mp) by CCR2^+^ CD64^+^ CD14^–^, Alveolar Mp by CD64^+^CD206^+^, dendritic cells (DC) by CD64^–^ CD206^–^ CD11c^+^ HLA-DR^+^, unidentified cells by CD64^–^ CD206^–^ CD11c^lo^ HLA-DR^–^. **(B)** The proportions of myeloid cell subpopulations in IPF, CTD-ILD, and sarcoidosis. Graphical plots represent individual samples. Statistical differences were analyzed by two-way ANOVA followed by Tukey’s multiple comparison test. n.s. not significant. **(C)** Citrus network tree visualizing the hierarchical relationship and intensity of each marker between identified myeloid cell populations gated by CD45^+^CD11b^+^ CD11c^+^ from IPF (n = 8), CTD-ILD (n = 11), and sarcoidosis (n = 10). Clusters with significant differences were represented in red, and those without significant differences in blue. Circle size reflects the number of cells within a given cluster. **(D)** Heatmap illustrating the expression markers across different clusters of myeloid cells as determined by the Citrus analysis. **(E)** Citrus-generated violin plots for eight representative and differentially regulated populations. Each cluster number (C#) corresponds to the number shown in panel **(C)**. All differences in abundance were significant at a false discovery rate < 0.01.

**Figure 3:**
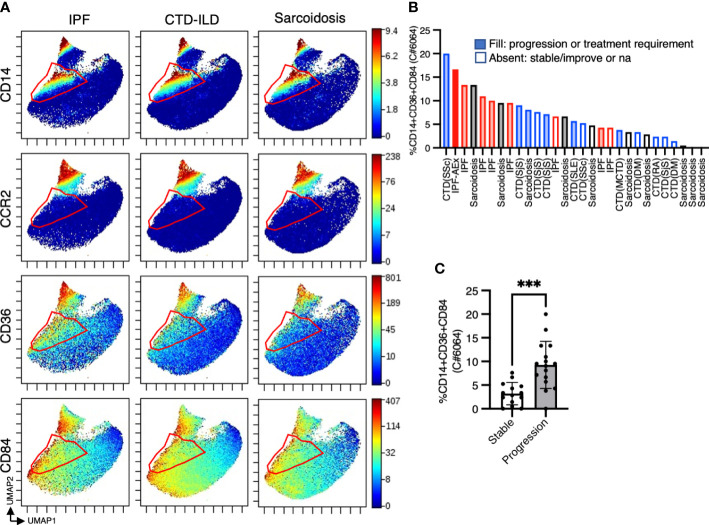
The proportion of CD14^+^CD36^hi^CD84^hi^ monocytes was correlated with disease progression. **(A)** UMAP plots showing CD14, CCR2, CD36, and CD84 expression in myeloid cells. Red triangles indicate CD14+ CCR2– cell subpopulations. **(B)** The proportion of CD14^+^ CD36^hi^ CD84^hi^ monocytes (cluster #6064 defined by the Citrus analysis in **Figure 2C**) abundance in myeloid cell populations in individual samples and **(C)** the correlation with disease progression. *** *p* < 0.001. Information for disease with clinical progression is also shown. IPF, idiopathic pulmonary fibrosis; CTD-ILD, connective-tissue disease-related interstitial lung disease; AEx, acute exacerbation; SSc, systemic sclerosis; SjS, Sjogren syndrome; SLE, systemic lupus erythematosus; RA, rheumatoid arthritis; DM, dermatomyositis; MCTD, mixed connective tissue disease; na, not accessed.

To investigate myeloid cell populations (identified as CD45^+^CD11b^+^CD11c^+^) that could provide insight into ILD conditions, we first conducted UMAP to see the major myeloid populations. The UMAP plot categorized 4 major subtypes, monocytes, CCR2+ macrophages, alveolar macrophages, dendritic cells, with no significant difference in IPF, CTD-ILD, and sarcoidosis (**Figures 2A, B**). We next utilized the Citrus algorithm to further investigate differently abundant myeloid cell subpopulations through the analysis of 18 parameters. Our analysis identified 33 clusters of myeloid cells, of which 23 were significantly differentiated between the groups (**Figure 2C** and **Supplementary Figure 2**). Clusters #6074, #6025, #6066, and #6054 were prevalent in sarcoidosis and characterized by CD64^+^ CD11b^lo^ CD14^–^ CD223(LAG3)^+^ HLA-DR^+^ CD163^hi^ expression (**Figures 2D, E**). Cluster #6059, which was abundant in CTD-ILD, was marked by CD64^+^ CD11c^+^ CD11b^+^ CD38^hi^ expression (**Figures 2D, E**). Clusters #6075, #6064, and #6056, which were prevalent in IPF, were comprised of CD64^+^ CD11b^hi^ CD11c^hi^ CD14^+^ CD36^hi^ CD84^hi^ CCR2^–^ monocyte subpopulations (**Figures 2D, E**), different from CD14^+^CCR2^+^ monocyte subpopulations (clusters #6078, #6082) (**Figures 2D**, **3A**). A recent report indicated that CD36^hi^ CD84^hi^ macrophages were increased in IPF compared to control and COPD lungs (28), which is consistent with our findings. Notably, the CD14^+^ CD36^hi^ CD84^hi^ CCR2^–^ monocyte subpopulation was also increased in ILDs with a progressive phenotype (**Figures 3B, C**), suggesting that these cells may have pathogenic properties.

### B cell subpopulations in the lungs of ILDs

**Figure 4:**
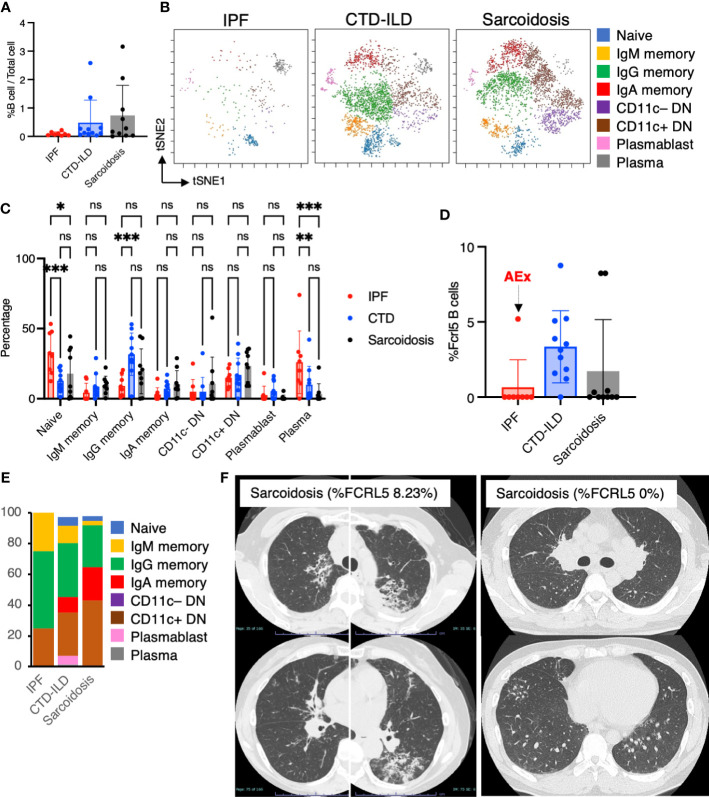
Characterization of B cell subsets in BALF from patients with IPF, CTD-ILD, and sarcoidosis. **(A)** Percentage of B cells and plasma cells in CD45^+^ BALF cells. **(B)** t-stochastic neighborhood embedding (t-SNE) plots of concatenated samples visualizing the distribution of B cell sub-populations in CD64^–^CD3^–^ and CD19^+^ or CD138^+^ gated B cells in BALF from patients with IPF, CTD-ILD, sarcoidosis. Naive B cells are defined by CD19^+^IgD^+^, IgM memory B cells: CD19^+^ IgM^+^ CD27^+^, IgG memory B cells: CD19^+^ IgG^+^ CD27^+^, IgA memory B cells: CD19^+^ IgA^+^ CD27^+^, CD11c^–^ double negative (DN) B cells: CD19^+^ CD11c^–^ IgD^–^ CD27^–^, CD11c^+^ DN B cells: CD19^+^ CD11c^+^ IgD^–^CD27^–^, plasmablasts: CD19^+^ CD27^+^ CD38^+^ CD138^–^, plasma cells: CD19^–^ CD138^+^ and IgG^+^ or IgA^+^. **(C)** Percentage of B cell sub-populations in IPF, CTD-ILD, and sarcoidosis. Graphical plots represent individual samples. **(D)** Percentage of FCRL5 expressing B cells in total B cells. ns: not significant, * *p* < 0.05, ** *p* < 0.01, *** *p* < 0.001. **(E)** The proportion of each B cell subsets in FCRL5 expressing B cells. **(F)** Representative chest CT images of patients with sarcoidosis exhibiting a high percentage of FCRL5 B cells and a low percentage of FCRL5 B cells in BALF.

Next, we sought to determine whether there were differential representations of B cell subsets in BALF cells of individuals with ILDs. Utilizing CD45^+^CD3^–^CD64^–^ and CD19^+^ or CD138^+^ as gating parameters, we were able to identify both B cells and plasma cells. It was observed that B cells tended to be more prevalent in individuals with CTD-ILDs and sarcoidosis compared to those with IPF, although the percentages of B cells/plasma cells remained low across all groups (**Figure 4A**). A t-SNE analysis of 17 parameters among B cells/plasma cells revealed the presence of various B cell subpopulations, including IgD^+^ naïve B cells, IgM^+^ memory B cells, IgG^+^ memory B cells, IgA^+^ memory B cells, IgD^–^CD27^–^ double negative (DN) B cells, plasmablasts, and plasma cells (**Figure 4B** and **Supplementary Figure 4**). This marked the first time that these B cell subpopulations were identified. The abundance of these subpopulations varied between groups, with particularly high levels of IgG memory B cells observed in individuals with CTD-ILDs compared to the other groups (**Figure 4C**).

Recent evidence indicated that CD11c^+^ DN B cells and FCRL5^+^ B cells are involved in the development of autoimmune conditions (16–18, 29). In this study, we, therefore, sought to examine these B cell subsets. While we did not observe any differences in CD11c^+^ DN B cells between individuals with IPF, CTD-ILDs, and sarcoidosis (**Figure 4C**), there was a tendency for FCRL5^+^ B cells to increase in CTD-ILDs relative to the other groups (**Figure 4D**). FCRL5^+^ B cells were mostly IgG^+^ or IgA^+^ in each group (IPF: 75%, CTD-ILD: 80.3%, Sarcoidosis: 91.7%) (**Figure 4E**), suggesting that these FCRL5^+^ B cells were class-switched B cells. Interestingly, 0% of FCRL5^+^ B cells were in all IPF cases except for one, which was AEx of IPF. The higher FCRL5^+^ B cell percentage in sarcoidosis was associated with progressive lung involvement (**Figure 4F**). Conversely, a patient with sarcoidosis exhibiting a low percentage of FCRL5^+^ B cells, bilateral hilar lymphadenopathy, and small nodules on CT (**Figure 4F**) demonstrated marked improvement on follow-up. These findings suggest that FCRL5^+^ B cells may possess pathogenic properties in CTD-ILDs as well as in the context of AEx of IPF and sarcoidosis.

### Characteristic T cell subpopulations in the lungs of ILDs

**Figure 5:**
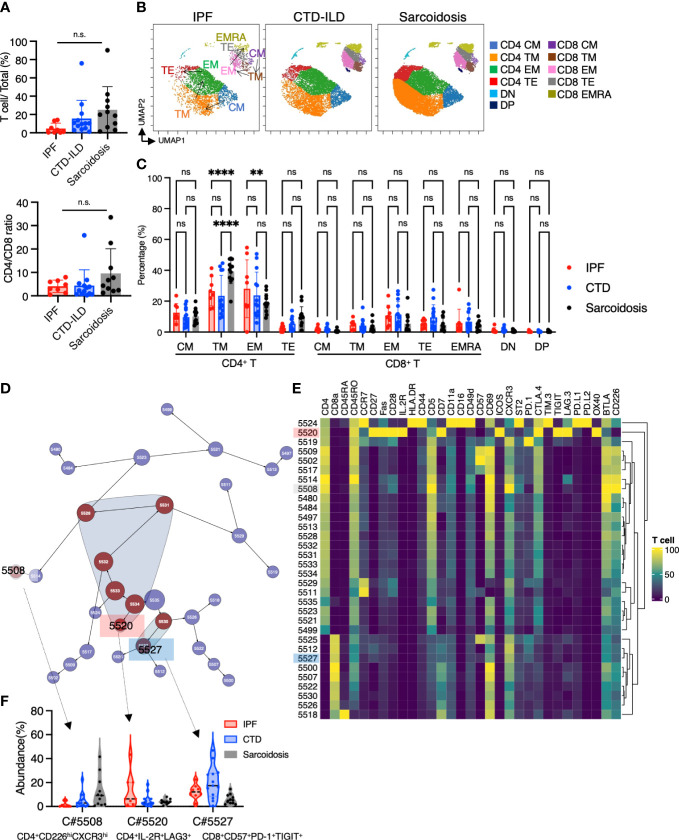
Characterization of T cell subsets in BALF from patients with IPF, CTD-ILD, and sarcoidosis. **(A)** The proportion of T cells (defined by CD2^+^CD3^+^) among CD45^+^ BALF cells and the CD4^+^ T cell/CD8^+^ T cell ratio from IPF (n = 8), CTD-ILD (n = 13), and sarcoidosis (n = 10). ns: not significant. **(B)** UMAP plots of concatenated samples visualizing the distribution of CD2^+^CD3^+^ T cell differentiation in BALF from patients with IPF, CTD-ILD, and sarcoidosis. Central memory (CM) T cells are defined by CCR7^+^ CD45RO^+^ CD28^+^ Fas^+^, Transitional memory (TM) by CCR7^–^ CD45RO^+^ CD28^+^ Fas^+^, Effector memory (EM) by CCR7^–^ CD45RO^+^ CD28^–^ Fas^+^, Terminal effector (TE) by CCR7^–^ CD45RO^+/–^ Fas^–^, Effector memory RA (EMRA) by CCR7^–^ CD45RO^–^ CD45RA^+^ Fas^+/–^. Arrows indicate the trajectory of T-cell differentiation. DN: CD4^–^ CD8^–^ double negative, DP: CD4^+^ CD8^+^ double positive. **(C)** Percentage of T cell subpopulations in IPF, CTD-ILD, and sarcoidosis. Graphical plots represent individual samples. Statistical differences were analyzed by two-way ANOVA followed by Tukey’s multiple comparison test. ns. not significant, ** *p* < 0.01, **** p < 0.0001. **(D)** The Citrus network tree displays the hierarchical relationship and intensity of each marker among the T-cell populations in BALF from IPF (n = 8), CTD-ILD (n = 13), and sarcoidosis (n = 10). **(E)** Heatmap illustrating the expression markers across different clusters of T cells as determined by the Citrus analysis. **(F)** Citrus-generated violin plots for three representative and differentially regulated populations. Each cluster number (C#) corresponds to the number shown in panel **(D)**. All differences in abundance are significant at a false discovery rate < 0.01.

In addition to myeloid cell and B cell subsets, we investigated whether subsets of T cells differentially existed in ILDs. The prevalence of T cells appeared to be elevated in sarcoidosis and CTD-ILDs compared to IPF, with the CD4/CD8 ratio tending towards an increase in sarcoidosis, although not reaching statistical significance (**Figure 5A**). To further visualize T cell differentiation within diseased lungs, we constructed UMAP plots (**Figure 5B**). BALF T cells primarily exhibited memory or effector phenotypes, with a scarcity of naive T cells (**Figures 5B, C**). Specifically, we observed a higher abundance of transient memory CD4 T cells in sarcoidosis and a higher abundance of effector memory CD4 T cells in IPF (**Figure 5C**). We next utilized the Citrus algorithm to distinguish differently abundant T cell subpopulations in IPF, CTD-ILD, and sarcoidosis in an unsupervised manner through analysis of 31 parameters (**Figure 5D** and **Supplementary Figure 5**). Our analysis identified 31 clusters of T cells, of which 9 were significantly differentiated between the groups. Cluster #5508, prevalent in sarcoidosis, was characterized by CD4^+^ CD226^+^ CXCR3^+^ (**Figures 5E, F**). Cluster #5520, prevalent in IPF, was comprised of CD4^+^ IL-2R^+^ TIGIT^+^ LAG3^+^, which are considered to be CD4^+^ regulatory T cells (Tregs) (**Figures 5E, F**) (6). Cluster #5527, which was abundant in CTD-ILDs, was marked by CD8^+^ CD57^+^ PD-1^+^ TIGIT^+^ (**Figures 5E, F**).

### Immunological phenotypes in a patient with acute exacerbation of IPF

**Figure 6:**
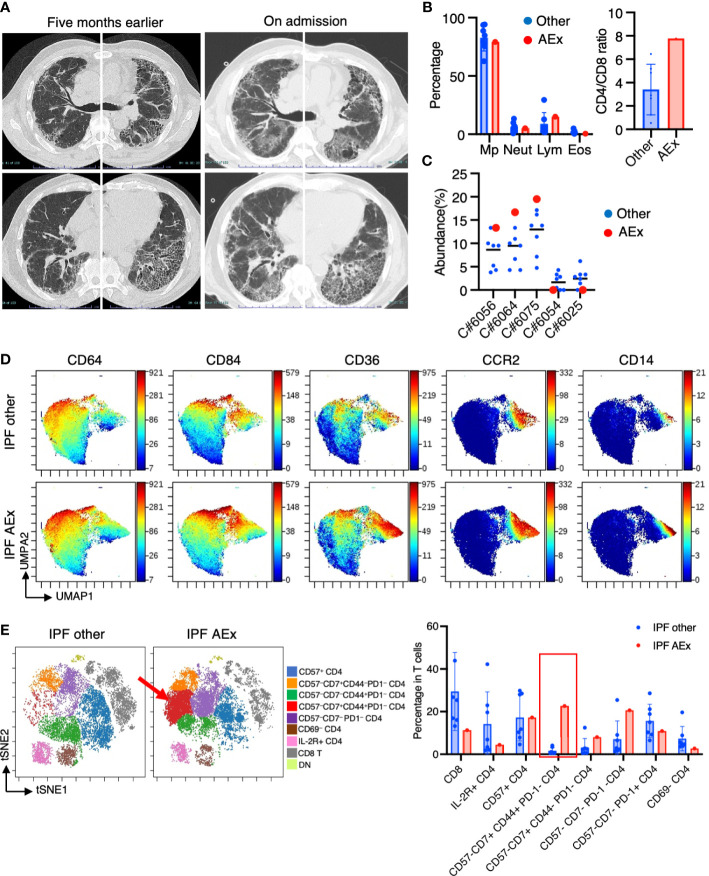
Immunological phenotypes in a patient with acute exacerbation of IPF. **(A)** Chest computed tomography images of the patient upon admission reveal the emergence of bilateral diffuse ground glass opacities superimposed on a honeycomb pattern, accompanied by peripheral traction bronchiectasis primarily in the basal lungs. **(B)** Comparison of BALF cell differentiation and CD4/CD8 ratio between patients with a patient experiencing an acute exacerbation (AEx) of the condition and the other cases of IPF. **(C)** Citrus-generated plots for myeloid sub-populations in IPF patients with stable condition and AEx. Each cluster number (C#) corresponds to the number shown in **Figure 2C**. **(D)** A uniform manifold approximation and projection (UMAP) of myeloid cells (CD45^+^CD11b^+^CD11c^+^ gated) showing cell distributions and each marker expression in BALF cells from concatenated samples with AEx and other cases of IPF. **(E)** t-SNE plots visualizing the distribution of T cell subpopulations in BALF T cells (gated as CD45^+^CD2^+^CD3^+^) from patients with AEx and other cases of IPF. Double negative (DN) T cells were defined as CD4^–^CD8^–^ T cells. A red arrow indicates expansion of the CD57-CD7+CD44+PD-1-CD4 T cell subpopulation in AEx-IPF.

One of the patients suffering from IPF experienced an acute exacerbation. The patient complained of worsening dyspnea upon exertion and a dry cough. Upon admission, chest CT images revealed the emergence of bilateral diffuse ground glass opacities superimposed on a honeycomb pattern, accompanied by peripheral traction bronchiectasis primarily in the basal lungs (**Figure 6A**). The patient’s blood procalcitonin, beta-D-glucan, and cytomegalovirus antigenemia were negative. The results of a PCR test for SARS-CoV-2 were also negative. BAL was performed for differential diagnosis, and a culture of the BAL fluid showed no presence of bacteria/mycobacteria. BALF cell differentiation showed a preponderance of macrophages (**Figure 6B**). The patient was then diagnosed with an acute exacerbation of IPF and treated with pulse methylprednisolone, tacrolimus, antibiotics, and recombinant thrombomodulin. Upon admission, he required 5 L/min of oxygenation at rest. He showed improvement, requiring only 0.5 L/min of oxygenation, and was discharged three weeks after admission. We compared mass cytometry analysis of cell subpopulations between AEx (n = 1) and other cases (n = 7) of IPF. The proportion of CD14^+^ CD36^hi^ CD84^hi^ CCR2^–^ monocyte populations (clusters #6056, #6064, #6075) in myeloid cells was highest in AEx compared to stable conditions in IPF (**Figure 6C**). A UMAP of myeloid cells showed increased CD36 and CD84 expression in AEx of IPF (**Figure 6D**). On the contrary, the proportions of myeloid clusters #6025 and #6054, characterized by CD64^+^ CD11b^lo^ CD14^–^ CD223 (LAG3)^+^ HLA-DR^+^ CD163^hi^ expression, were lowest in AEx of IPF (**Figure 6C**). t-SNE analysis of T cells showed a decreased proportion of CD8^+^ T cells and an increased proportion of CD4^+^ CD57^–^ CD7^+^ CD44^+^ PD1^–^ subsets in AEx of IPF (**Figure 6E**).

### Immunological phenotypes in BALF cells from patients with CTD-ILD

**Figure 7:**
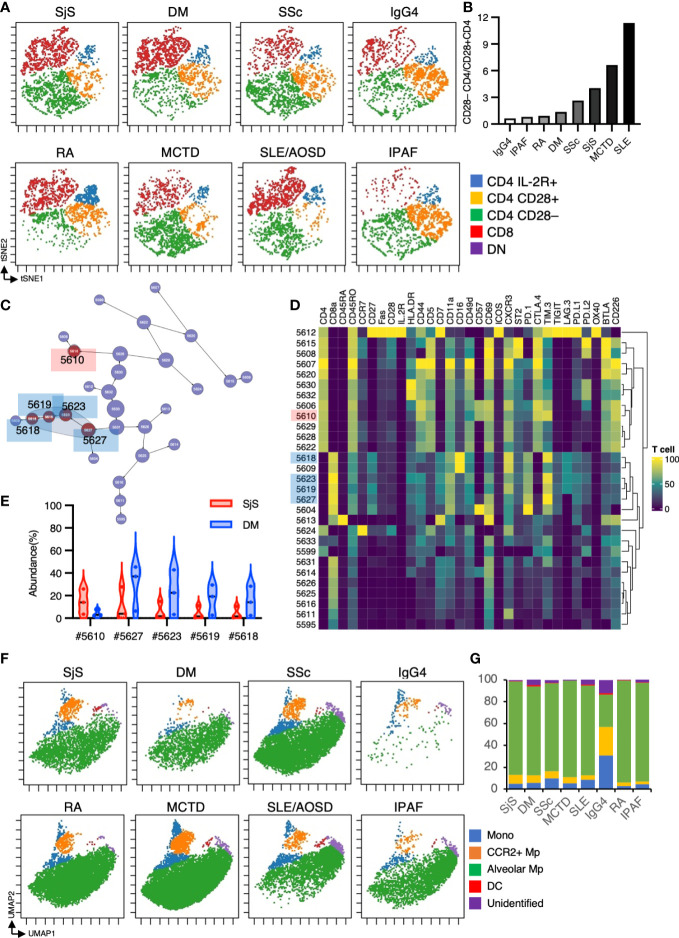
Immunological phenotypes in BALF cells from patients with CTD-ILD. **(A)** t-SNE plots visualizing the distribution of T cell subpopulations in BALF T cells (gated as CD45^+^CD2^+^CD3^+^) from patients with CTD-ILD. DN: CD4–CD8– double negative T cells. **(B)**The ratio of CD28^–^ CD4 T cells/CD28^+^ CD4 T cells defined in t-SNE plots in each disease. **(C)** The Citrus network tree displays the hierarchical relationship and intensity of each marker among the T-cell populations in BALF from Sjogren syndrome-related ILD (n = 3) and dermatomyositis associated-ILD (n = 3). **(D)** Heatmap illustrating the expression markers across different clusters of T cells as determined by the Citrus analysis. **(E)** Citrus-generated violin plots for five representative and differentially regulated populations. Each cluster number (C#) corresponds to the number shown in panel **(C)**. All differences in abundance are significant at a false discovery rate < 0.01. **(F)** UMAP plots visualizing the distribution of CD11b^+^CD11c^+^ myeloid cell subpopulations in BALF from patients with CTD-ILD. Monocytes are defined by CD64^+^CD14^+^, CCR2^+^ macrophages (Mp) by CCR2^+^ CD64^+^ CD14^–^, Alveolar Mp by CD64^+^CD206^+^, dendritic cells (DC) by CD64^–^ CD206^–^ CD11c^+^ HLA-DR^+^, unidentified cells by CD64^–^ CD206^–^ CD11c^lo^ HLA-DR^–^. **(G)** The proportions of myeloid cell subpopulations in CTD-ILD.

CTD-ILD encompasses diverse diseases that may exhibit divergent immune cell abnormalities. Hence, we have compared each CTD-ILD, that is, SjS, DM, SSc, RA, SLE, MCTD, IgG4-related, and IPAF. **Figures 7A, B** show that the CD28^–^CD4/CD28^+^CD4 ratio was significantly divergent between the diseases, with the highest ratio from SLE-related ILDs. Previous research indicated that CD28^–^ CD4^+^ T cells were prevalent in the blood of SLE patients with nephritis (30). These CD28– CD4+ T cells were infiltrated in the renal tissue and may have contributed to renal injury (30). Given this prior report, we speculated that these CD28^–^ CD4^+^ T cells might also play a pathological role in SLE-related ILDs. Subsequently, we performed a CITRUS analysis on T cell subsets between SjS and DM (the CITRUS analysis can be conducted if the sample size is three or more.) (**Figures 7C–E**). The CITRUS analysis revealed that TIM-3^hi^ CD8+ T cells were more abundant in patients with DM compared to those with SjS, which is concordant with the findings from prior publications (31, 32).

In the myeloid subset, a lower proportion of alveolar macrophages with higher monocytes and CCR2^+^ macrophages were observed in IgG4-related ILDs (**Figures 7F, G**). Nevertheless, due to the very limited number of cases in each group, it is challenging to conclude whether the disparities in cell populations are intrinsic differences or not.

## Discussion

We have here demonstrated the characteristic immune cell subpopulations present in BALF from patients with IPF, CTD-ILDs, and sarcoidosis. Our analysis revealed an expansion of CD14^+^CD36^hi^CD84^hi^ CCR2^–^ monocytes in patients with IPF, an increase in FCRL5^+^ B cell in patients with CTD-ILDs and AEx of IPF, increased levels of IL-2R^+^ TIGIT^+^ LAG3^+^ CD4^+^ T cells in IPF, increased levels of CXCR3^+^ CD226^+^ CD4^+^ T cells in sarcoidosis, and increased levels of PD1^+^ TIGIT^+^ CD57^+^ CD8^+^ T cells in CTD-ILDs.

CD36 is a scavenger receptor expressed on the surface of immune and non-immune cells that acts as a signaling receptor for damage-associated molecular patterns (DAMPs) and pathogen-associated molecular patterns (PAMPs) and also serves as a transporter for long-chain free fatty acids (33). CD84 is an immunoreceptor expressed on the surface of various immune cells that regulates a range of immunological processes, including T cell cytokine secretion, natural killer cell cytotoxicity, monocyte activation, autophagy, T–B interactions, and B cell tolerance at the germinal center checkpoint (34). Ayaub et al. recently reported that CD36^hi^ CD84^hi^ macrophages were expanded as a specific subpopulation of macrophages in the lungs of patients with IPF compared to healthy or chronic obstructive pulmonary disease lungs using single-cell RNA-sequencing, mass cytometry, and flow cytometry (28). Ayaub et al. demonstrated that these CD36^hi^ CD84^hi^ macrophages expressed both alveolar and interstitial lung macrophage markers (HLA-DR^+^, CD11b^+^, CD206^+^), which is consistent with our results. Importantly, our study demonstrated these macrophage populations could be detected from BALF samples, which is less invasive compared to a lung biopsy and more suitable for clinical applications. We further determined that these CD36^hi^ CD84^hi^ macrophages were CD14 positive, indicating that these cells originated from monocytes.

We discovered these CD36^hi^ CD84^hi^ monocyte subpopulations were highest in AEx of IPF among all IPF cases. Nakashima et al. demonstrated that pulmonary fibrosis led to significant alterations in the bone marrow, including the expansion and activation of monocytic cells, which enhanced fibrosis upon subsequent lung injury (35). The pathobiology of IPF AEx is likely triggered by an acute event that leads to widespread acute lung injury, along with the acceleration of underlying chronic factors contributing to the fibrotic process (23). From this evidence, we speculate that these CD36^hi^ CD84^hi^ monocyte subpopulations may be involved in accelerating the AEx of IPF. Interestingly, these CD36^hi^CD84^hi^ CD14^+^CCR2^–^ monocyte populations were ST2^+^ and CCR5^+^ but distinct from CCR2^+^ monocyte populations (**Figures 2D**, **3A**). Liang et al. demonstrated that the expression of CCL2, a ligand for CCR2, suppressed bleomycin-induced pulmonary fibrosis in mice (36). In addition, it has been shown that CCR2 deficiency in a mouse model of silica-induced pulmonary fibrosis resulted in an expansion of the fibrotic area (37), suggesting that CCR2^hi^ monocyte-derived macrophages may have a suppressive role in fibrosis. Increased monocyte count in blood samples has been identified as a cellular biomarker for poor outcomes in fibrotic diseases, including IPF (38). A more detailed analysis of subsets of monocytes in the blood may be more useful for predicting the prognosis of ILDs.

FCRL5, encoded by the immunoglobulin superfamily receptor translocation-associated 2 (*IRTA2)* gene, is a member of the Fc receptor-like family, and its expression is mainly restricted to B cells. It has nine extracellular immunoglobulin domains, two immunoreceptor tyrosine-based inhibitory motifs, and one presumed immunoreceptor tyrosine-based activation motif in its cytoplasmic tail (39). FCRL5 signaling, in conjunction with B cell receptor activation and TLR9 engagement, can lead to B-cell proliferation, activation, isotype switching, and the production of IgG- and IgA-positive B cells (29). Higher FCRL5 expression predicted response to rituximab in rheumatoid arthritis (40) and granulomatosis with polyangiitis and microscopic polyangiitis (16). There are reports that single nucleotide variants in the FCRL5 gene increase an individual’s predisposition to multiple sclerosis (41) or SLE (42). This evidence indicates the pathogenic role of FCRL5 on autoimmunity. This study demonstrated increased FCRL5^+^ B cells in CTD-ILDs and a case of AEx of IPF. A recent study has demonstrated that rituximab was not inferior to cyclophosphamide in treating patients with CTD-ILDs with fewer adverse events (43). Collectively, higher FCRL5^+^ B cells in BALF could be used as a biomarker as a rationale for using rituximab.

We observed higher levels of IL-2R^+^ CD4^+^ T cells in patients with IPF compared to those with CTD-ILDs and sarcoidosis. These IL-2R^+^ CD4^+^ T cells likely represent Tregs, given that IL-2R^+^ expressing CD4+ T cells are a sole, distinct T cell subpopulation characterized by the highest FOXP3 expression between T cell subsets (6). In the study by Serezani et al. (6), the proportions of CD4 Tregs were a higher tendency in IPF compared to controls. Increased activated Treg proportion was correlated with the severity of IPF (44). However, the precise roles of Tregs in the development of pulmonary fibrosis have not been fully elucidated, and the existing literature on Tregs in pulmonary fibrosis is inconsistent (45). On the one hand, Tregs may contribute to the progression of pulmonary fibrosis by secreting platelet-derived growth factor (PDGF), transforming growth factor-β (TGF-β), and other factors that promote epithelial-mesenchymal transition and alter the Th1/Th2 balance. On the other hand, Tregs may inhibit fibrosis by promoting the repair of epithelial cell damage, inhibiting the accumulation of fibroblasts, and strongly suppressing the production and function of pro-inflammatory factors and cells. The roles of Treg would, thus, be dependent on the time and microenvironment in regard to pulmonary fibrosis. We would like to note that these Tregs express PD-L1. We previously reported the PD-L1 expression in T cells from BALF and the emergence of PD-1 and PD-L1 co-expressing T cells in particular situations, such as a severe immune-checkpoint inhibitor-related ILD (46) and adult T cell leukemia-affected lungs (47). These PD-L1 expressing T cells, including CD4 T cells, may have an immune-suppressing function on neighboring effector T cells or a *cis-*regulatory fashion on itself (48) *via* PD-1/PD-L1 interaction.

We showed increased levels of CXCR3^+^ CD226^+^ CD4^+^ T cells in sarcoidosis. CXCR3 ligands, CXCL9 and CXCL11, are augmented in BALF from pulmonary sarcoidosis (49). These CXCL9 and CXCL11 are interferon-inducible chemokines and localized to epithelioid histiocytes and multinucleated giant cells forming non-necrotizing granulomas (49). Our observation matches the results of the previous study and suggests that CXCR3 ligands may recruit CXCR3-expressing CD4 T cells, which propagate a type 1 immune response and cause granuloma formation. CD226, also known as DNAM-1, is an adhesion molecule that plays a role in activating T cells through the interaction with CD115 and CD112 as ligands (50), competing for TIGIT as reciprocal functions. This is the first report to mention CD226 expression in T cells from sarcoidosis. We speculate that CD4 T cells migrating to the lungs through CXCR3 may be activated through CD226, potentially contributing to the pathogenesis of sarcoidosis.

CD57 expression divided T cell subpopulations in viSNE. CD57 expression is typically associated with NK cells, but it has also been found in T cells that have a more advanced differentiation state, reduced replicative capacity, and increased production of cytokines such as IFNγ (51–53). There is also some evidence that CD57^+^ CD8^+^ T cells may have cytotoxic functions and be correlated with autoimmune activity in type 1 diabetes (54). We found increased levels of CD57^+^CD8^+^PD-1^+^TIGIT^+^ subsets in CTD-ILDs, and these CD8+ subsets may be related to an autoimmune condition.

Our study has several limitations, such as the lack of data from healthy controls, the relatively small sample size, and the retrospective design, which resulted in missing clinical data for some cases. Selection biases may be present, as only patients who underwent the BAL procedure could enroll in this study. In summary, our study demonstrates different immune cell phenotypes in IPF, CTD-ILDs, and sarcoidosis. We discovered that CD14^+^CD36^+^CD84^+^ monocytes and FCRL5^+^ B cells, important immune subsets, are differentially expressed and may be pathogenic. Further studies based on these findings may result in the development of new cell-targeted strategies to inhibit ILD progression.

## Data availability statement

The original contributions presented in the study are included in the article/**Supplementary Material**. Further inquiries can be directed to the corresponding author.

## Ethics statement

The studies involving human participants were reviewed and approved by the Ethics Committee of Kyushu University Hospital. Written informed consent for participation was not required for this study in accordance with the national legislation and the institutional requirements.

## Author contributions

Conceptualization: TY; Methodology: KH, TY, KM, KK, and YB; Investigation: KH, TY, KM, KK, KS, KT, DE, HA, and MU; Visualization: KH, TY, KM, KK, KS, KT, DE, HA, MU, SI, and YB; Funding acquisition: TY and YF; Supervision: SI, YB, YF, and IO; Writing-original draft: KH and TY; Writing-review and editing: SI, YB, YF, and IO. All authors contributed to the article and approved the submitted version.
