# Supplementary material for: Mass cytometry identifies characteristic immune cell subsets in bronchoalveolar lavage fluid from interstitial lung diseases
Source: Front Immunol. 2023 Mar 6;14:1145814. doi: 10.3389/fimmu.2023.1145814 (PMC10027011; doi:10.3389/fimmu.2023.1145814)
Supplement: Supplementary file 1 [file DataSheet_1.docx]

Supplementary information

**Mass cytometry identifies characteristic immune cell subsets in bronchoalveolar lavage fluid from interstitial lung diseases**

Kentaro Hata^1,4^, Toyoshi Yanagihara^1,4*^, Keisuke Matsubara^2^, Kazufumi Kunimura^2^,

Kunihiro Suzuki^1^, Kazuya Tsubouchi^1^, Daisuke Eto^1^, Hiroyuki Ando^1^, Maki Uehara^1^, Satoshi Ikegame^1^, Yoshihiro Baba^3^, Yoshinori Fukui^2^, Isamu Okamoto^1^

^1^ Department of Respiratory Medicine, Graduate School of Medical Sciences, Kyushu University, Fukuoka, Japan

^2^ ﻿Division of Immunogenetics, Department of Immunobiology and Neuroscience, Medical Institute of Bioregulation, Kyushu University, Fukuoka, Japan

^3^ Division of Immunology and Genome Biology, Department of Molecular Genetics, Medical Institute of Bioregulation, Kyushu University, Fukuoka, Japan

^4^ These authors contributed equally to this work and share the first authorship

^*^ **Corresponding author:** Toyoshi Yanagihara

E-mail: yanagihara.toyoshi.759@m.kyushu-u.ac.jp

**Supplemental Table 1. Mass cytometry antibody panels.**

For CD45 barcoding

| **Label** | **Target** | **Product_id** | **Clone** | **Amount (uL)** |
| --- | --- | --- | --- | --- |
| 89Y | CD45 | 3089003B | HI30 | 1 |
| 106Cd | CD45 | 3106001C | HI30 | 1 |
| 110Cd | CD45 | 3110001C | HI30 | 1 |
| 112Cd | CD45 | 3112001C | HI30 | 1 |
| 116Cd | CD45 | 3116001C | HI30 | 1 |
| 196Pt | CD45 | 3196001C | HI30 | 1 |

For antibody cocktail #1 (T cell panel)

| **Label** | **Target** | **Product ID** | **Clone** | **Amount (uL)** |
| --- | --- | --- | --- | --- |
| 141Pr | CD3 | 3141019B | UCHT1 | 1 |
| 142Nd | CD11a | 3142006B | HI111 | 0.5 |
| 143Nd | CD5 | 3143007B | UCHT2 | 1 |
| 145Nd | CD4 | 3145001B | RPA-T4 | 1 |
| 146Nd | CD8a | 3146001B | RPA-T8 | 1 |
| 147Sm | CD7 | 3147006B | CD7-6B7 | 1 |
| 148Nd | CD274 (PD-L1) | 3148017B | 29E.2A3 | 1 |
| 149Sm | CD25 (IL-2R) | 3149010B | 2A3 | 1 |
| 150Nd | CD134 (OX40) | 3150023B | ACT35 | 1 |
| 151Eu | CD2 | 3151003B | TS1/8 | 1 |
| 152Sm | CD95/Fas | 3152017B | DX2 | 1 |
| 153Eu | TIM-3 | 3153008B | F38-2E2 | 1 |
| 154Sm | TIGIT | 3154016B | MBSA43 | 1 |
| 155Gd | CD279 (PD-1) | 3155009B | EH12.2H7 | 1 |
| 156Gd | CD183 (CXCR3) | 3156004B | G025H7 | 1 |
| 158Gd | ST2 (R&D AF523) | Labeling (201158A) |  | 1 |
| 159Tb | CD197 (CCR7) | 3159003A | G043H7 | 1 |
| 160Gd | CD28 | 3160003B | CD28.2 | 1 |
| 161Dy | CD152 (CTLA-4) | 3161004B | 14D3 | 1 |
| 162Dy | CD69 | 3162001B | FN50 | 1 |
| 163Dy | CD272 (BTLA) | 3163009B | MIH26 | 1 |
| 164Dy | CD45RO | 3164007B | UCHL1 | 1 |
| 165Ho | CD223/LAG-3 | 3165037B | 11C3C65 | 1 |
| 166Er | CD44 | 3166001B | BJ18 | 1 |
| 167Er | CD27 | 3167002B | O323 | 1 |
| 168Er | CD278/ICOS | 3168024B | C398.4A | 1 |
| 169Tm | CD19 | 3169011B | HIB19 | 1 |
| 170Er | CD45RA | 3170010B | HI100 | 1 |
| 171Yb | CD226 | 3171013C | DX11 | 1 |
| 172Yb | CD273 (PD-L2) | 3172014B | 24F.10C12 | 1 |
| 173Yb | HLA-DR | 3173005B | L243 | 0.5 |
| 174Yb | CD49d | 3174018B | 9F10 | 1 |
| 175Lu | CD14 | 3175015B | M5E2 | 2 |
| 176Yb | CD57 | 3176019B | HCD57 | 1 |
| 209Bi | CD16 | 3209002B | 3G8 | 1 |

For antibody cocktail #2 (B cell/ myeloid cell panel)

| **Label** | **Target** | **Product ID** | **Clone** | **Amount (uL)** |
| --- | --- | --- | --- | --- |
| 141Pr | CD3 | 3141019B | UCHT1 | 1 |
| 142Nd | CD19 | 3142001B | HIB19 | 0.5 |
| 143Nd | CD11b (Biolegend #301302) | Labeling(201143A) | ICRF44 | 0.5 |
| 144Nd | CD38 | 3144014B | HIT2 | 1 |
| 145Nd | CD163 | 3145010B | GHI/61 | 1 |
| 146Nd | CD64 | 3146006B | 10.1 | 1 |
| 147Sm | CD11c | 3147008B | Bu15 | 0.5 |
| 148Nd | IgA | 3148007B | Polyclonal | 1 |
| 149Sm | IgG (Biolegend #410701) | Labeling(201149A) |  | 1 |
| 150Nd | CD138 | 3150012B | DL-101 | 0.5 |
| 151Eu | CD209 (Biolegend #343002) | Labeling(201209A) |  | 1 |
| 152Sm | CD21 | 3152010B | BL13 | 1 |
| 153Eu | CD192 (CCR2) | 3153023B | K036C2 | 1 |
| 154Sm | CD84 | 3154013B | CD84.1.21 | 1 |
| 156Gd | CD86 | 3156008B | IT2.2 | 1 |
| 158Gd | ST2 (R&D AF523) | Labeling (201158A) |  | 1 |
| 159Tb | CD36 (Biolegend #336215) | Labeling(201159A) | 5-271 | 1 |
| 160Gd | CD28 | 3160003B | CD28.2 | 0.5 |
| 164Dy | CD185/CXCR5 | 3164029B | RF8B2 | 1 |
| 165Ho | CD223/LAG-3 | 3165037B | 11C3C65 | 1 |
| 166Er | CD24 | 3166007B | ML5 | 1 |
| 167Er | CD27 | 3167002B | O323 | 1 |
| 168Er | CD206 (MMR) | 3168008B | 44607 | 1 |
| 169Tm | CD32 | 3169020B | FUN-2 | 1 |
| 170Er | TIM-1 (Biolegend #353902) | Labeling(201170A) |  | 1 |
| 171Yb | CD195 (CCR5) | 3171017A | NP-6G4 | 1 |
| 172Yb | IgM | 3172004B | MHM-88 | 1 |
| 173Yb | HLA-DR | 3173005B | L243 | 0.5 |
| 174Yb | IgD (Biolegend #348235) | Labeling(201174A) | IA6-2 | 1 |
| 175Lu | CD14 | 3175015B | M5E2 | 2 |
| 176Yb | antiAPC-176Yb | 3176007B | APC003 | 1 |
| 209Bi | CD16 | 3209002B | 3G8 | 1 |

**Supplementary Table 2. Cell count for each case.**

| FCS Filename | T panel | | Myeloid/B panel | | |
| --- | --- | --- | --- | --- | --- |
|  | Total cell | T cell | Total cell | Myeloid cell | B cell |
| CTD SSc #1 | 63035 | 8570 | 84466 | 60350 | 232 |
| CTD MCTD | 102002 | 9931 | 156572 | 123954 | 170 |
| CTD SjS #1 | 121862 | 13329 | 116147 | 95750 | 138 |
| CTD SjS #2 | 94705 | 3558 | 25574 | 17518 | 32 |
| CTD DM #1 | 21480 | 1616 | 146025 | 108897 | 54 |
| CTD DM #2 | 3011 | 939 | 3276 | 1784 | 15 |
| CTD SLE | 50027 | 2542 | 53443 | 37405 | 30 |
| STD SjS #3 | 37127 | 7790 | 43953 | 26406 | 1136 |
| CTD DM #3 | 95330 | 1117 | 81012 | 29461 | 247 |
| CTD IgG4 | 13966 | 10597 | 15786 | 2148 | 100 |
| CTD RA | 80401 | 2355 | 84384 | 74096 | 211 |
| CTD IPAF | 38350 | 3937 | 43091 | 27449 | 38 |
| CTD SSc #2 | 5971 | 622 | 5834 | 4441 | 80 |
| IPF #1 | 23944 | 3169 | 22006 | 16153 | 49 |
| IPF #2 | 85676 | 2559 | 80571 | 48529 | 23 |
| IPF #3 | 28320 | 1501 | 34312 | 25305 | 15 |
| IPF #4 | 100285 | 1130 | 94206 | 47110 | 96 |
| IPF #5 | 55994 | 213 | 50295 | 40666 | 40 |
| IPF #6 | 41030 | 204 | 37288 | 28952 | 15 |
| IPF #7 | 34782 | 173 | 29711 | 27248 | 46 |
| IPF #8 | 99579 | 13929 | 116187 | 90957 | 77 |
| Sarcoidosis #1 | 7233 | 2539 | 8519 | 2732 | 9 |
| Sarcoidosis #2 | 31964 | 6350 | 55463 | 37983 | 92 |
| Sarcoidosis #3 | 99552 | 22669 | 70378 | 42589 | 22 |
| Sarcoidosis #4 | 13712 | 835 | 19810 | 7452 | 626 |
| Sarcoidosis #5 | 132968 | 55816 | 84433 | 44594 | 242 |
| Sarcoidosis #6 | 9603 | 2724 | 6689 | 3612 | 41 |
| Sarcoidosis #7 | 44160 | 39798 | 41496 | 2623 | 850 |
| Sarcoidosis #8 | 71337 | 1041 | 76737 | 71921 | 0 |
| Sarcoidosis #9 | 43464 | 1345 | 29030 | 25567 | 9 |
| Sarcoidosis #10 | 63692 | 11934 | 85865 | 62076 | 814 |

**Supplementary Table 3. Clinical features of CTD-ILD.**

| **CTD** | **Auto-antibodies** | **Radiological patterns** |
| --- | --- | --- |
| SjS #1 | SS-A, CENP-B | UIP |
| SjS #2 | SS-A, SS-B | NSIP |
| SjS #3 | SS-A, SS-B, RNA pol III | LIP |
| DM #1 | MDA5 | NSIP |
| DM #2 | ARS | NSIP |
| DM #3 | ARS | NSIP |
| SSc #1 | CENP-B | UIP |
| SSc #2 | CENP-B | NSIP |
| RA | CCP, MPO-ANCA | UIP |
| MCTD | RNP | NSIP |
| SLE | Sm, SS-A | Unclassifiable |
| IgG4 | - | OP |
| IPAF | SS-A | NSIP |

The case of IPAF was diagnosed with the criteria below.

1. Presence of interstitial pneumonia (by HRCT): ○
2. Exclusion of alternative aetiologies: ○
3. Does not meet criteria of a defined connective tissue disease: ○
4. At least one feature from at least two of these domains: ○
5. Clinical domain: ×
6. Serologic domain: ○ (SS-A)
7. Morphologic domain: ○ (NSIP pattern by HRCT)

**Supplementary Figure 1. PCA analysis of myeloid cells and T cells in BALF cells from IPF, CTD-ILD, and sarcoidosis.**

Scatter plots from total cells, myeloid cells, and T cells were found to be similar within a given disease group in IPF, while scatter plots from total cells and myeloid cells in sarcoidosis and T cells in CTD-ILD were found to be dissimilar within their respective disease groups. Note that the scatter plots from each group were different but not completely separated, with some overlap observed.


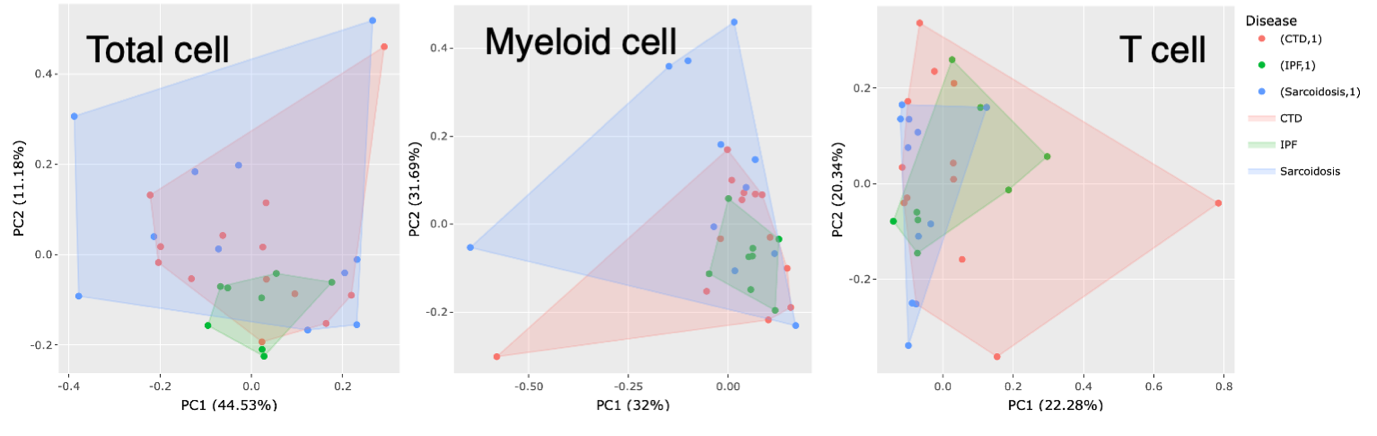


**Supplementary Figure 2. Citrus analysis of myeloid cell populations in BALF cells from IPF, CTD-ILD, and sarcoidosis.**

**
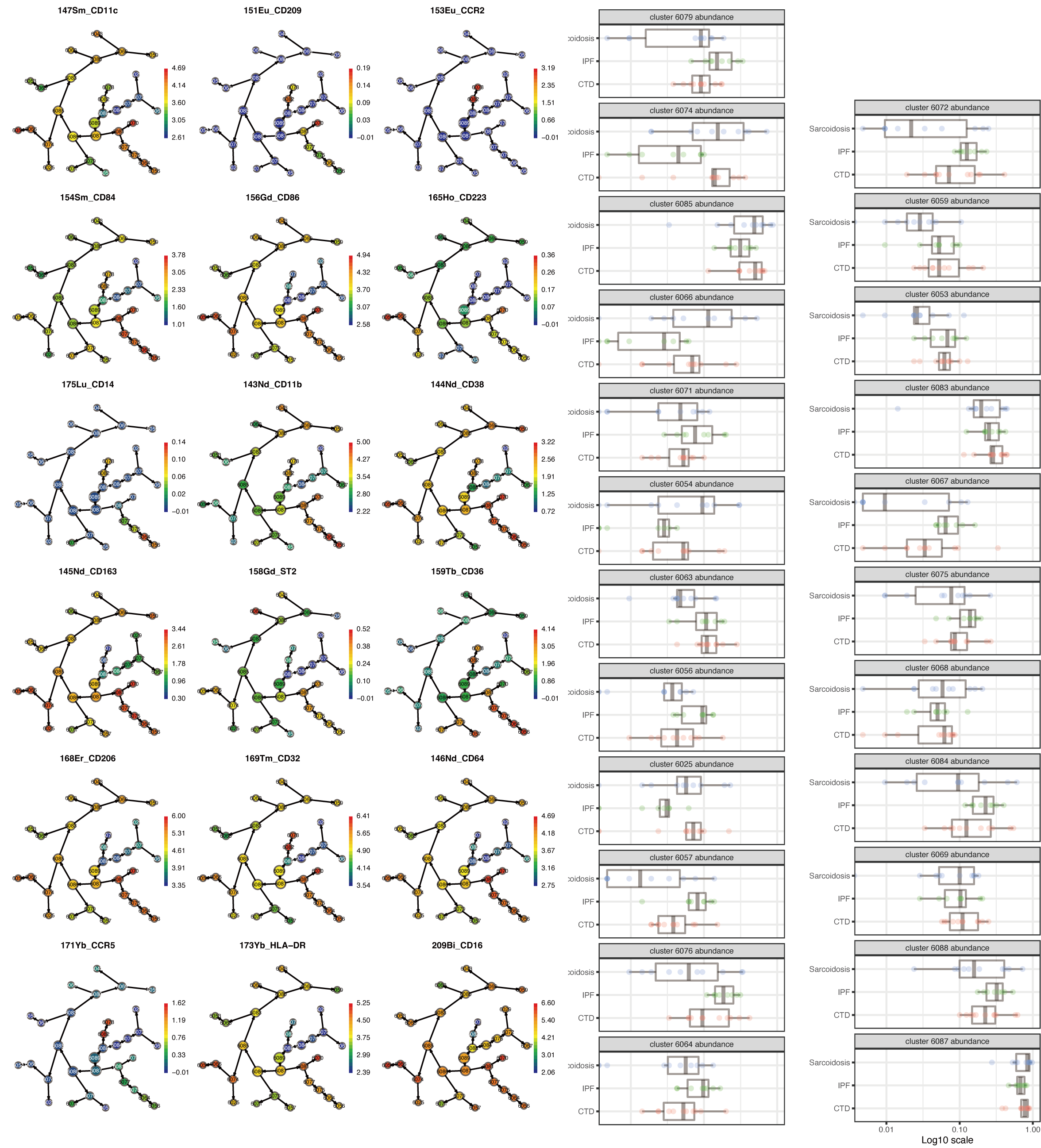
**

**Supplementary Figure 3. UMAP of concatenated samples visualizing distribution of myeloid cell sub-populations in BALF from patients with IPF, CTD-ILD, and sarcoidosis.**

**
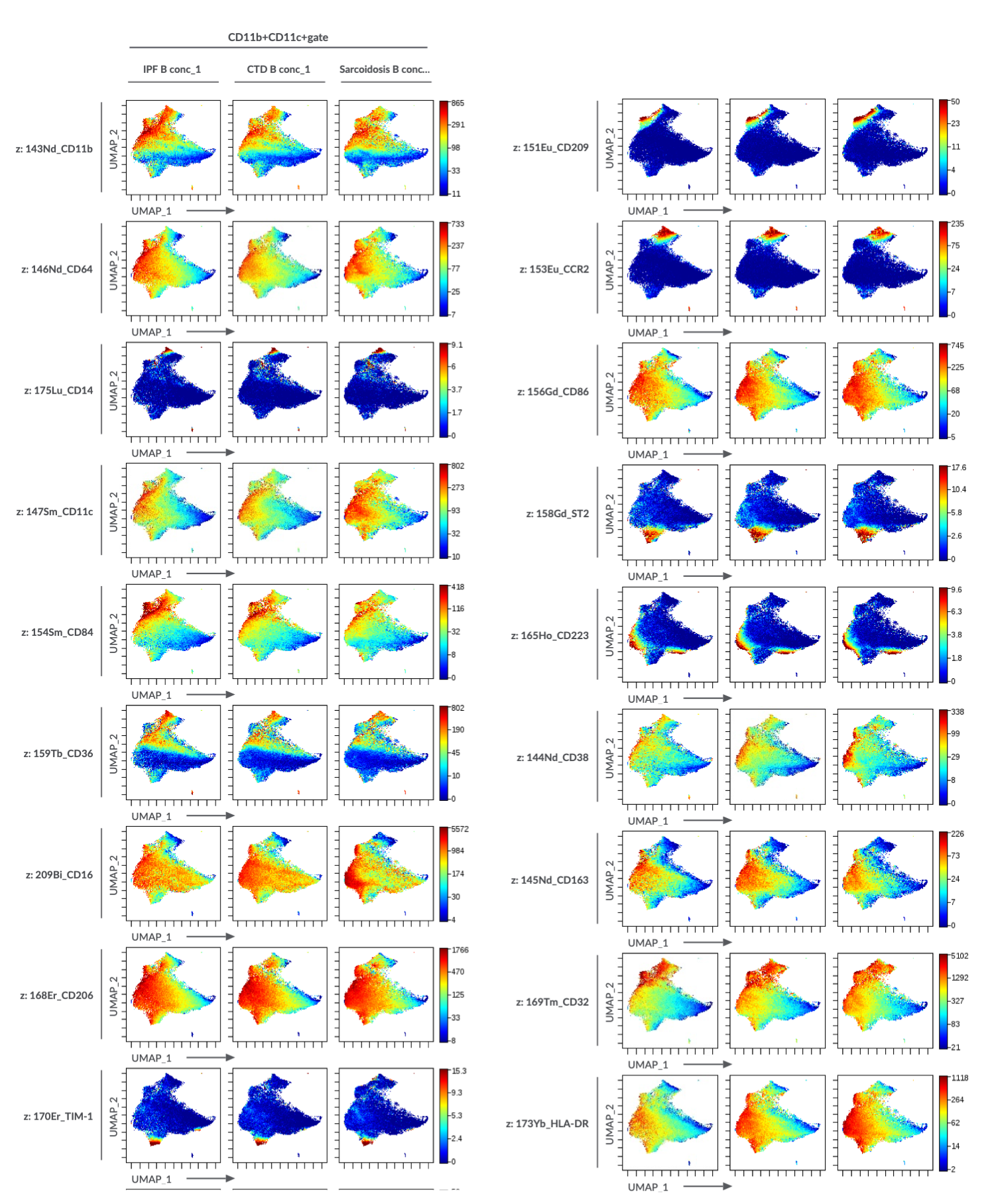
**

**Supplementary Figure 4. t-stochastic neighborhood embedding (t-SNE) plots of concatenated samples visualizing distribution of B cell sub-populations in BALF from patients with IPF, CTD-ILD, sarcoidosis.**

**
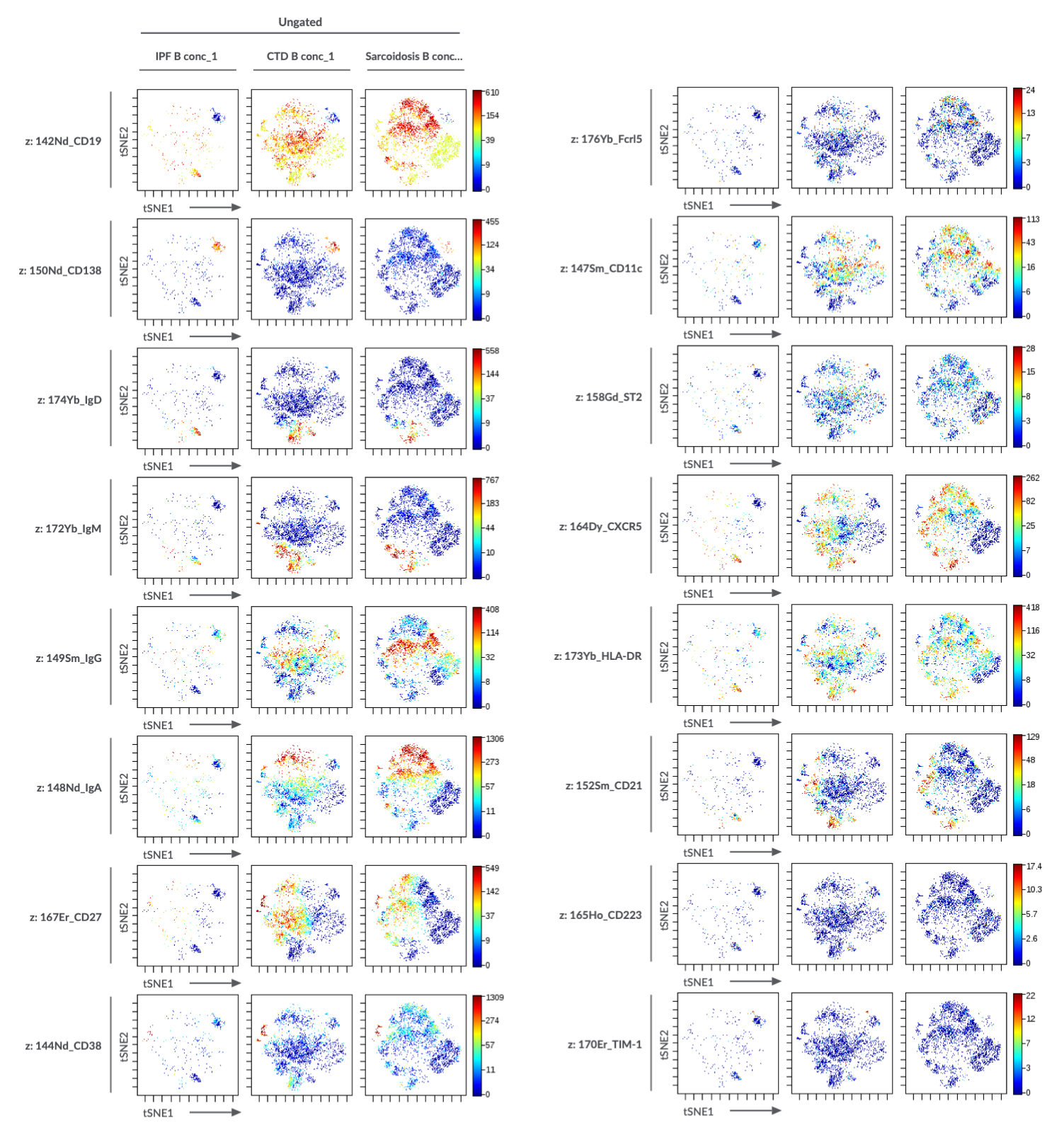
**

**Supplementary Figure 5. A T cell gate (CD2^+^CD3^+^) and Citrus analysis of T cell populations in BALF cells from IPF, CTD-ILD, and sarcoidosis.**

**
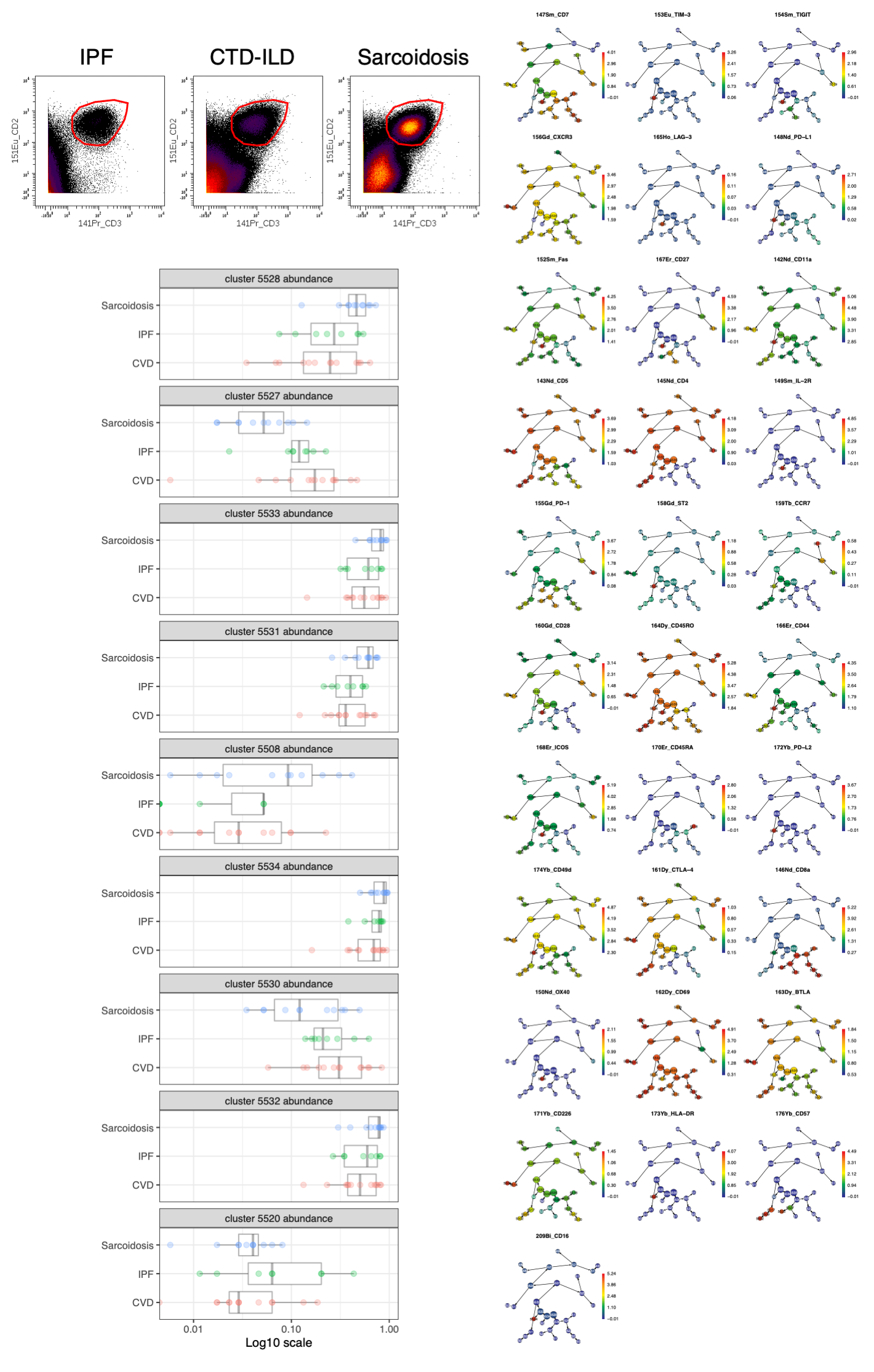
**

**Supplementary Figure 6. t-stochastic neighborhood embedding (t-SNE) plots of concatenated samples visualizing distribution of T cell sub-populations in BALF from patients with IPF, CTD-ILD, sarcoidosis.**

**
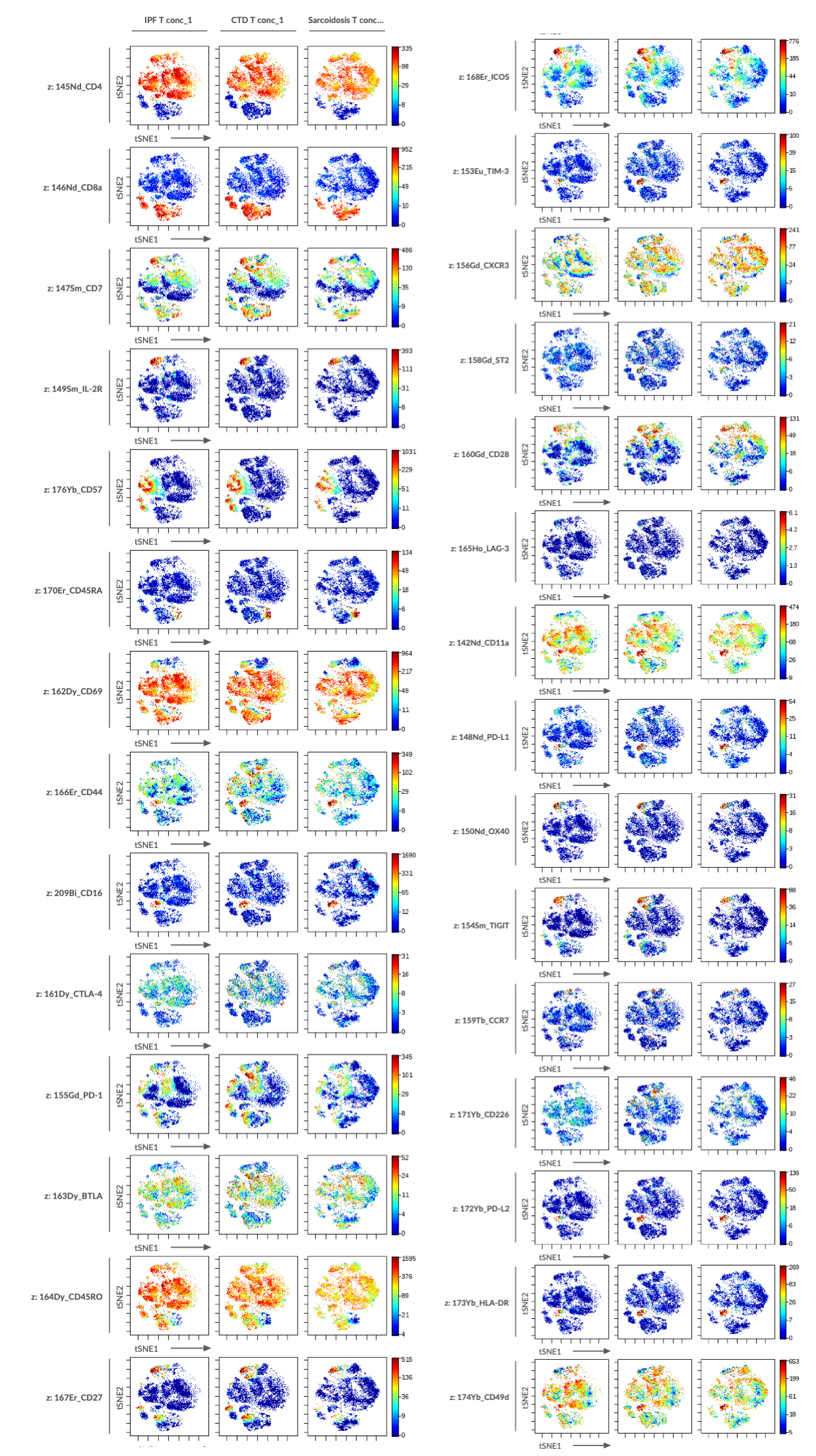
**
